# A Broad Distribution of the Alternative Oxidase in Microsporidian Parasites

**DOI:** 10.1371/journal.ppat.1000761

**Published:** 2010-02-12

**Authors:** Bryony A. P. Williams, Catherine Elliot, Lena Burri, Yasutoshi Kido, Kiyoshi Kita, Anthony L. Moore, Patrick J. Keeling

**Affiliations:** 1 School of Biosciences, Geoffrey Pope Building, University of Exeter, Exeter, Devon, United Kingdom; 2 Department of Botany, University of British Columbia, Vancouver, British Columbia, Canada; 3 Department of Biochemistry and Biomedical Sciences, School of Life Sciences, University of Sussex, Falmer, Brighton, United Kingdom; 4 Department of Biomedical Chemistry, Graduate School of Medicine, University of Tokyo, Tokyo, Japan; University of California Los Angeles, United States of America

## Abstract

Microsporidia are a group of obligate intracellular parasitic eukaryotes that were considered to be amitochondriate until the recent discovery of highly reduced mitochondrial organelles called mitosomes. Analysis of the complete genome of *Encephalitozoon cuniculi* revealed a highly reduced set of proteins in the organelle, mostly related to the assembly of iron-sulphur clusters. Oxidative phosphorylation and the Krebs cycle proteins were absent, in keeping with the notion that the microsporidia and their mitosomes are anaerobic, as is the case for other mitosome bearing eukaryotes, such as *Giardia*. Here we provide evidence opening the possibility that mitosomes in a number of microsporidian lineages are not completely anaerobic. Specifically, we have identified and characterized a gene encoding the alternative oxidase (AOX), a typically mitochondrial terminal oxidase in eukaryotes, in the genomes of several distantly related microsporidian species, even though this gene is absent from the complete genome of *E. cuniculi*. In order to confirm that these genes encode functional proteins, AOX genes from both *A. locustae* and *T. hominis* were over-expressed in *E. coli* and AOX activity measured spectrophotometrically using ubiquinol-1 (UQ-1) as substrate. Both *A. locustae* and *T. hominis* AOX proteins reduced UQ-1 in a cyanide and antimycin-resistant manner that was sensitive to ascofuranone, a potent inhibitor of the trypanosomal AOX. The physiological role of AOX microsporidia may be to reoxidise reducing equivalents produced by glycolysis, in a manner comparable to that observed in trypanosomes.

## Author Summary

Microsporidia are obligate intracellular parasites responsible for a number of diseases in commercially important animals (e.g. bees) and of significant medical concern, in particular in immunocompromised humans. Though related to fungi, microsporidia have undergone a rapid phase of adaption to the intracellular environment and have in the process reduced many aspects of their biology. Notably, microsporidia have highly reduced mitochondria (powerhouses of the cell) reflected in reduced energy metabolic pathways. Thus they likely produce ATP only through the process of glycolysis. In some parasites, this glycolytic pathway is dependent on an additional step involving a protein called the “alternative oxidase”. We have shown that this protein is also present in several species of microsporidia. Crucially, this protein is absent from humans and so can potentially be exploited as a drug target. Our experiments show that this protein is likely widespread in microsporidia, and is sensitive to the antibiotic ascofuranone, which is currently being tested as a potential treatment for the agent causing sleeping sickness. Our results suggest that knowledge gleaned from drug trials on sleeping sickness is potentially transferrable to the treatment of some cases of microsporidiosis.

## Introduction

Microsporidia are a large and diverse group of eukaryotic intracellular parasites that infect a wide variety of animal lineages, including humans [1]. Although once thought to be early branching eukaryotes, they are now widely accepted to be very atypical parasitic fungi [2],[3],[4],[5]. They are highly adapted to the infection process, and many typical eukaryotic features have been simplified, reduced, or lost completely. Microsporidian genomes are reduced and organelles such as the peroxisome, mitochondria and Golgi apparatus are absent or altered from their canonical forms [6],[7],[8].

In particular, microsporidian mitochondria have been severely reduced into biochemically and physically streamlined “mitosomes” [8]. Mitosomes lack their own genome, and there is no evidence from the nuclear genome of any microsporidian for genes encoding any of the respiratory chain complexes or an F_1_-ATP synthase complex. In the absence of the ability to synthesize ATP through oxidative phosphorylation, microsporidia appear to import ATP directly from their host cell via ATP translocases located in the cell membrane [9],[10], using a transporter which may have been acquired by lateral gene transfer from bacterial energy parasites such as *Chlamydia* and *Rickettsia* [11]. Identification of which mitochondrial-derived genes have been retained in the complete genome of *Encephalitozoon cuniculi*, together with immunolocalization studies in *E. cuniculi* and *Trachipleistophora hominis*, suggest that the major functional role for the mitosome is not in energy generation, but instead the assembly of iron-sulphur clusters for export to the cytoplasm [6],[12],[13].

Biochemical and genomic evidence all generally point to glycolysis as the major route of energy generation in most microsporidia [6],[9]. In order for ongoing glycolytic activity to be sustainable, however, some mechanism to reoxidise reducing equivalents produced by this pathway is also required. Of the few proteins associated with the microsporidian mitosomes that are not involved in iron-sulfur cluster assembly, one is glycerol-3-phosphate dehydrogenase. This enzyme is the mitochondrial component of the glycerol-3-phosphate shuttle, a pathway used in some eukaryotes to move reducing equivalents into mitochondria [14]. Both cytosolic and mitochondrial components of this shuttle are encoded in the genomes of several microsporidia that have been well studied [6],[15], and it has been suggested that this could provide a mechanism sustaining glycolysis in the cytosol by reoxidising glycerol-3-phosphate [9]. However, the *E. cuniculi* mitochondrial glycerol-3-phosphate dehydrogenase does not appear to be located in the mitochondrion any longer [12], and even if a working shuttle was present, there is no obvious mechanism for reoxidation of the co-reduced FAD produced by this shuttle in the genome of *E. cuniculi* [6]. In the bloodstream form of *Trypanosoma brucei* parasites, the mitochondrial glycerol-3-phosphate dehydrogenase is coupled to an alternative oxidase (AOX) that together achieve this process [16], and a similar system has been postulated to be present in the apicomplexan parasite *Cryptosporidium parvum* [17].

AOX is a cyanide-insensitive terminal oxidase that is typically located on the inner surface of the inner mitochondrial membrane. It branches from the main respiratory chain at the level of the ubiquinone pool, results in the net reduction of oxygen to water, and is non-protonmotive [18],[19],[20]. It has been found in some prokaryotic lineages, including alpha-proteobacteria [21], and has a wide but discontinuous distribution across eukaryotes: it is widely distributed in plants, and has also been found in a handful of invertebrate animals [22],[23],[24],[25]. In parasitic protists, the distribution of AOX is also uneven: it is known from the amoebozoan *Acanthamoeba castellanii*, the heterokont *Blastocystis hominis*, and the trypanosomes. In the alveolates, it is found in the apicomplexan *Cryptosporidium* and some other distantly related alveolates including some ciliates, but absent from the more closely related *Plasmodium* parasites [26],[27],[28]. The broad overall distribution of AOX may be indicative of an early origin in eukaryotes, and is perhaps even derived from the endosymbiotic alpha-proteobacterium that gave rise to mitochondria [27],[29].

In fungi, the protein also has a wide but discontinuous distribution [30], but it is absent from the completely sequenced genome of *E. cuniculi* and from the recent large-scale genome surveys of *Nosema ceranae* and *Enterocytozoon bieneusi* [6],[31],[32]. Interestingly, however, we identified a homologue in the partially sequenced genome of *Antonospora locustae*, demonstrating the pattern of retention versus loss is also uneven within the microsporidia, despite their otherwise common mode of intracellular parasitism and apparently similar metabolism. The possible presence and function of AOX in microsporidia is of practical interest as well, because the absence of AOX in mammals, including humans, renders it a potential therapeutic target for the treatment of microsporidiosis, as is the case in a number of protisitan parasites [16],[33],[34]. This is of particular importance in microsporidia as current medical treatments are not universally effective. The drugs of choice for microsporidiosis are currently albendazole and fumagillin [35]. Whilst albendazole is used in treating many species, some, such as *V. corneum* and *E. bieneusi* are resistant and in these cases fumagillin, which is mildly toxic, has to be used [36].

Here, we characterize the phylogenetic distribution of microsporidian AOX, and examine the functional activity of AOX enzymes from the human parasite *T. hominis* and the insect parasite *A. locustae*. Phylogenetically the microsporidian AOX is weakly related to mitochondrial homologues from other eukaryotes, and both *A. locustae* and *T. hominis* AOX proteins include an N-terminal leader that was demonstrated by confocal microscopy to target the proteins to mitochondria in yeast, altogether suggesting the enzyme is likely derived from the mitosome and may be localized in the organelle still, though direct co-localization would be required to give a definitive location of function. Enzyme assays with recombinant proteins demonstrated both possess cyanide-resistant oxidase activities sensitive to inhibition by the very specific trypanosome AOX inhibitor ascofuranone [37], suggesting the enzyme functions as a terminal electron receptor.

## Results

### AOX is broadly distributed in microsporidian parasites

The complete genome of *E. cuniculi* lacks any gene resembling AOX, but we identified a full-length homologue of the AOX gene in the partial genome of *A. locustae* (gmod.mbl.edu/perl/site/antonospora01, *Antonospora locustae* Genome Project, Marine Biological Laboratory at Woods Hole, funded by NSF award number 0135272). To determine the distribution of this gene, degenerate PCR was used to amplify a short fragment of AOX from other species of microsporidia, *Glugea plecoglossi* (233 bp), *Spraguea lophii* (235 bp), and *T. hominis* (236 bp). To examine the complete sequence of an AOX from a human parasite, the ends of the *T. hominis* AOX gene were also sequenced using 5′ RACE and splinkerette protocols [38], resulting in a full length gene of 957 bp with a translated protein of 318 amino acids (compared to *A. locustae* AOX, which had a length of 831 bp).

**Figure 1:**
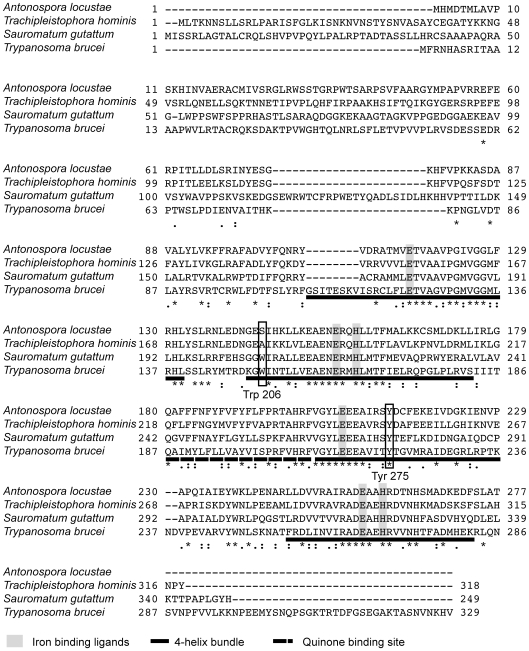
Alignment of *A. locustae, T. hominis*, *S. guttatum* and *T. brucei* AOX sequences. The four-helix bundles are underlined with a solid line. The putative quinone binding site is underlined with a broken line. Conserved amino acid sites are marked with a star and semi conserved sites are marked with dots.

Hypothetical translations of *A. locustae* and *T. hominis* sequences contain all conserved sites consistent with AOX activity. Specifically, both genes encode the six conserved di-iron binding ligands that are essential for AOX activity (Figure 1), which are conserved in all alternative oxidases sequenced to date [17],[20],[39]. In addition both sequences contain 4 highly conserved tyrosine residues, one of which (Tyr at the *S. guttatum* equivalent position 275) is considered to be critical for the net reduction of oxygen to water and probably plays a key role in enzyme catalysis (Figure 1) [19]. Further confirmation that *A. locustae* and *T. hominis* sequences encode AOX proteins is the finding that a putative substrate binding site (residues 242–263) [19] is also conserved in both microsporidia. However, one striking difference between the microsporidian AOX sequences and those AOX sequences found in all other mitochondria and protists is the lack of tryptophan-206, which is most unusual since it is highly conserved and has been proposed to play either a structural or catalytic role [18]. In *A. locustae* the tryptophan has been replaced by serine whilst in *T. hominis* it has been replaced by alanine. Similar to other parasite AOXs however, none of the cysteines postulated to play a role in the regulation of AOX activity in plants [40], are present in either *A. locustae* or *T. hominis*.

**Figure 2:**
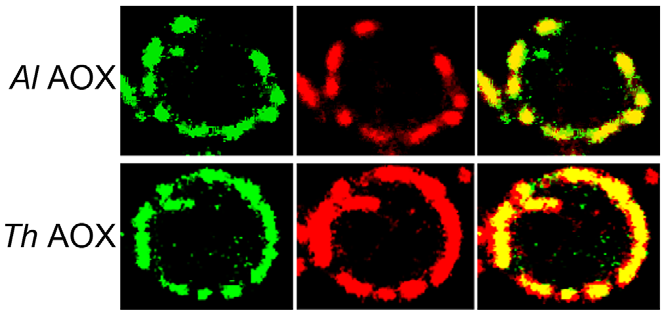
Transfection of *S. cerevisiae* cells with AOX-GFP constructs. The left panel represents the signal from the GFP-AOX constructs, the centre image represents the mitotracker signal accumulated in the yeast mitochondria. The right panel shows the composite image of the GFP and the red mitotracker signal. The top set (ThAOX) represents the *T. hominis* AOX-GFP construct, the lower set (AlAOX) represents the *A. locustae* AOX-GFP construct. In each set of panels a single yeast cell is shown with the branched mitochondrial network around the periphery of the cell that has an approximate diameter of 6 µm.

Mitoprot I predicted both microsporidian AOX sequences to encode amino-terminal mitochondrial transit peptides, and the *T. hominis* AOX protein was also predicted by Predotar and TargetP 1.1 to have a mitochondrial targeting peptide. In order to test the degree of conservation and functionality of potential targeting signals, full-length proteins were expressed in *S. cerevisiae* cells fused to a green fluorescent reporter protein. Expression in yeast shows that GFP overlays mitotracker fluorescence, indicating successful heterologous targeting for both proteins (Figure 2).

### Phylogenetic analysis

**Figure 3:**
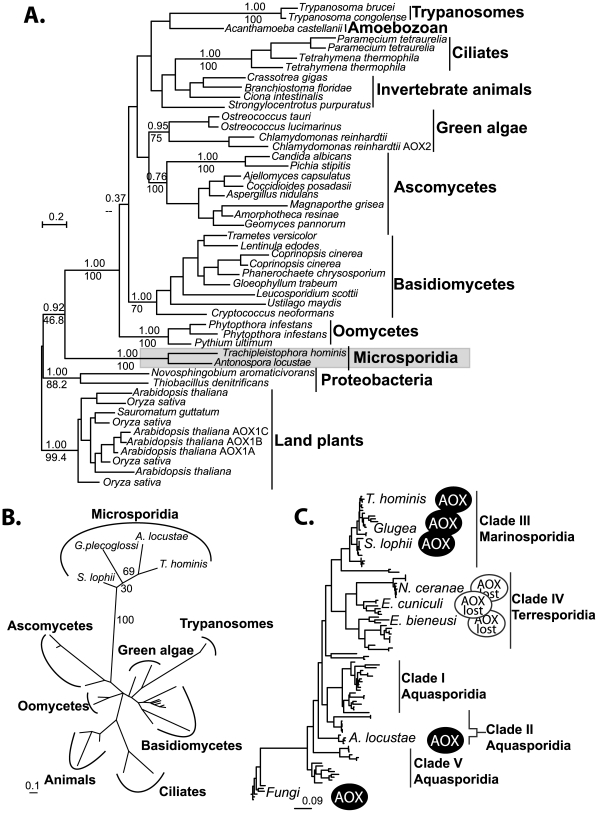
Phylogenetic analyses of microsporidian AOX sequences. **A.** Global MrBayes AOX phylogeny, posterior probabilities and PhyML bootstraps from an analysis of 500 bootstrapped datasets are shown above and below respectively, key and well supported clades (>70% Bootstrap). **B.** Short alignment PhyML phylogeny including the translated amplified sequences from all four microsporidia. Bootstrap support from 100 datasets is shown next to microporidian nodes. **C.** Microsporidian distribution of the alternative oxidase gene plotted onto a phylogeny of microsporidian SSU rDNA sequences. Scale bars in all trees indicate substitutions per site.

The phylogenetic relationship among alternative oxidases is in general poorly resolved. There are several well-supported clades, including the microsporidia, the ascomycete fungi, and the basidiomycete fungi, but the fungi do not form a single well-supported clade (Figure 3A), similar to results recovered in earlier AOX phylogenies [27]. The strong support uniting AOX from *A. locustae* and *T. hominis* does, however, confirm the microsporidian genes share a single common origin. Phylogenetic analysis based on the conserved region of the gene amplified from other microsporidia similarly places *S. lophii* and the *G. plecoglossi* in the same monophyletic microsporidian group (Figure 3B), further supporting the common origin of all microsporidian AOX genes. The overall distribution of microsporidian AOX was therefore mapped onto an SSU phylogeny including all major clades of microsporidia as defined by molecular and ecological data [41], which showed that AOX is widely distributed in microsporidia, and perhaps only absent from a single clade of predominantly vertebrate and insect parasites, including *E. cuniculi*, *E. bieneusi* and *N. cerenae* (Figure 3C).

### Structure and function of microsporidian AOX proteins

**Figure 4:**
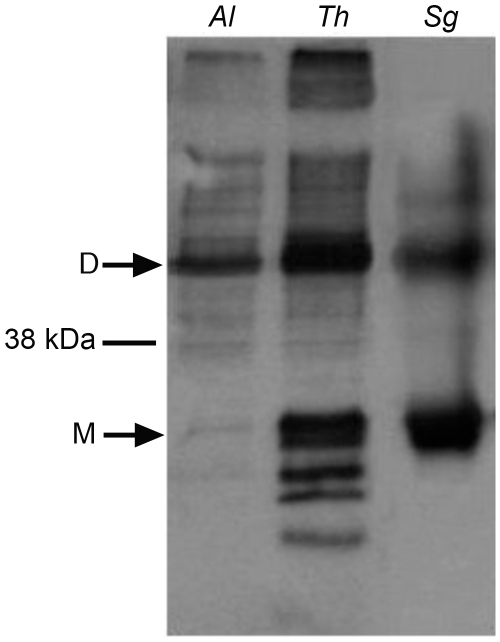
Western blots of the membrane preparations from C41 *E. coli* strains expressing microsporidian AOX proteins. Lane 1 shows *A. locustae* rAOX and Lane 2 shows *T. hominis* rAOX. Lane 3 shows purified alternative oxidase protein from *Sauromatum guttatum*.

To directly examine the function of *A. locustae* and *T. hominis* AOX proteins (especially given the sequence difference reported in Figure 1), recombinant *A. locustae* and *T. hominis* AOX proteins were expressed in *E. coli* and the enzyme structure and activity was measured. Antibodies raised against the plant AOX recognize both *A. locustae* and *T. hominis* proteins (Figure 4), and both a monomer and a dimer can be detected in Western blots of non-reducing gels, as is the case within the thermogenic plant *Sauromatum guttatum*, although in the case of *A. locustae* the monomer is not very prominent. (Figure 4). In *E. coli* membrane fractions containing either *A. locustae* or *T. hominis* recombinant AOX (rAOX), ubiquinol-1 oxidase activity indicates that the activities of both proteins are as expected for AOX (Table 1). In both cases, 1 µM antimycin A, 2 µM myxothiazol and 1 mM potassium cyanide were included in the assay system to ensure inhibition of the cytochrome *bo* and *bd* complexes of *E. coli*, and the specific activities reported in Table 1 have been corrected for auto-oxidation of ubiquinol-1 in the absence of membranes (see methods). It is important to note that, although *A. locustae* rAOX was more active than *T. hominis* rAOX, both proteins were equally sensitive to 10 nM ascofuranone (Table 1), the very specific and potent inhibitor of the trypanosomal alternative oxidase [37]. Furthermore, it is apparent from Table 1 that the specific activities of these microsporidia are considerably higher than those reported for rAOX from *C. parvum* [17] but comparable to those observed with overexpression studies of *T. brucei* rAOX in *E. coli* membranes [42].

**Table 1:** Rates of oxidation of 150 µM ubiquinol-1 by these membrane fractions.

Sample	Specific Activity µmol QH2 oxidised min^-1^ mg^-1^
*A. locustae* rAOX in *E. coli* membranes	2.2
+10 nM ascofuranone	0.2
*T. hominis* rAOX in *E. coli* membranes	1.6
+10 nM ascofuranone	0.1
*C. parvum* rAOX in *E. coli* membranes [17]	0.03

## Discussion

The genome of *E. cuniculi* has served as a model for microsporidian metabolism since it was completed [6], however, it has never been clear how this model organism dealt with the reducing potential built up through ongoing glycolysis, since it lacks a terminal oxidase. Here we show that this model does not reflect microsporidia as a whole, because alternative oxidase has a broad distribution amongst microsporidian parasites. This distribution remains discontinuous, however, because we can say with some confidence that AOX is not present in either the *E. cuniculi* or *N. ceranae* genomes, which have been sequenced to near completion [6],[32]. It also appears to be absent from the genome of *E. bieneusi*, although this genome is not completely sampled [31]. Our negative PCR results from *E. aedis* and *A. (Brachiola) algerae* are less conclusive (these have previously been shown to have a high AT content that may prevent the successful amplification of the AOX gene by degenerate PCR [43]), but it suggests the gene may also be absent in several other lineages. Whilst *G. plecoglossi*, *T. hominis* and *S. lophii* are quite closely related and within the Marinosporidia clade, *Antonospora locustae* falls within the distantly related Aquasporidia clade as defined by molecular and ecological analysis [41] (Figure 3C). As we know that the alternative oxidase is present in at least two major clades, and in many fungi, the most parsimonious explanation for its distribution in microsporidia is that it was present in their last common ancestor, but has been lost in *E. cuniculi* and probably other lineages during their more recent evolutionary history.

Analysis of the AOX sequences from *A. locustae* and *T. hominis* reveals that both possess the iron-and substrate-binding motifs found in other AOXs. In *S. guttatum*, Tyr-253 has been shown to be involved in substrate binding, and Tyr-275 to be critical for catalytic activity [19],[44], and both of these are also conserved in microsporidia. The absences of Trp-206 in *A. locustae* and *T. hominis* AOX sequences is somewhat surprising, as it is conserved across all other known mitochondrial AOX sequences. Since *A. locustae* and *T. hominis* AOX sequences are demonstrably functional (Table 1), Trp-206 cannot play a universally critical role in electron transport, but it may have a role in other mitochondrial AOXs as helping to anchor the protein to the leaflet of the inner mitochondrial membrane in a manner seen with other monotopic membrane proteins [19],[20],[45].

The demonstration that *A. locustae* and *T. hominis* rAOX have a high quinol oxidase activity that is sensitive to ascofuranone at nanomolar concentrations not only solves a significant puzzle in microsporidian metabolism, but also offers a new avenue of treatment for some microsporidian species and further “in tissue culture” trials can establish the efficiency of the drug across the life cycle of the microsporidian. There is currently considerable interest in this antibiotic, originally isolated from the phytopathogenic fungus *Ascochyta visiae*, for its potential promise in the treatment of trypanosomiasis and cryptosporidiosis. The fact that it also appears to potently inhibit the microsporidian AOX may give the drug a more widespread use than previously considered. Of course several of the microsporidia that parasitise humans lack the AOX (e.g. *E. cuniculi* and *E. bieneusi*), but for other human parasites (e.g. *T. hominis*) the AOX is clearly a potential target, and may also be in other unexplored lineages (e.g., *Vittaforma corneae*).

With respect to the potential function of AOX in microsporidia a possible role may be similar to that proposed in the bloodstream form of some trypanosomes. In the bloodstream form of *Trypanosoma brucei*, where glucose is abundant and there is no conventional respiratory chain [16], ATP synthesis is switched from oxidative phosphorylation to substrate level phosphorylation. Glycolysis is contained within a glycosome, a membrane-bound organelle containing glycolytic enzymes. In this system, reducing equivalents generated by glycolysis in the form of glycerol-3-phosphate are shuttled out of the glycosome and re-oxidised by a glycerol-3-phosphate dehydrogenase (G3PDH) located on the outer surface of the inner membrane. G3PDH itself reduces the mitochondrial ubiquinone pool that in turn is then re-oxidised by the alternative oxidase. In this way, glycerol-3-phosphate within the glycosome is continuously being re-oxidised to supply further substrate for the net oxidation of NADH [16]. Thus in an indirect manner mitochondrial alternative oxidase activity maintains the NADH/NAD balance within the glycosomes. In addition to the alternative oxidase, however, trypanosomes also possess a glycerol kinase that under anaerobic conditions helps to maintain the glycosome NADH/NAD balance by converting glycerol-3-phosphate to glycerol [16].

**Figure 5:**
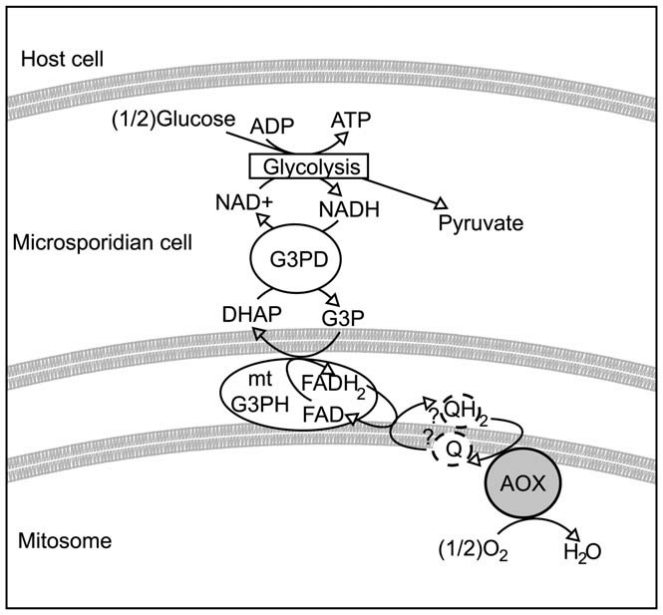
Hypothetical scheme of function of the alternative oxidase in the microsporidian cell. Microsporidian cells are known to contain glycolytic enzymes, though no obvious mechanism exists for reoxidising NADH to NAD+. The glycerol-3-phosphate shuttle is encoded in many microsporidian genomes. If this shuttle is coupled to an alternative oxidase protein in the mitosome, it could potentially represent a mechanism for regenerating NAD+.

It is plausible that most microsporidia rely on a similar system and that AOX fulfils the role of the terminal oxidase, as shown in Figure 5. Whether the microsporidian AOX functions in the mitosome or cytosol is not completely certain, but its very presence in the cell and its carrying out the functions we have demonstrated in vitro significantly change our view of microsporidian metabolism and drug sensitivity in either event. Overall, the presence of an N-terminal leader with characteristics of a transit peptide, together with the likely mitochondrial origin of the protein, all suggest a mitosomal location is most plausible. This also fits well with previously unusual observations on the glycerol-3-phosphate shuttle. Localization studies on mitochondrial glycerol-3-phosphate dehydrogenase in *E. cuniculi* show no evidence that the enzyme is confined to mitochondria or specifically localized there, unlike ferredoxin, frataxin, ISCU and ISCS [12],[13], and in *E. bieneusi* the gene appears to be absent altogether [31]. This suggests that the glycerol shuttle has been displaced in these microsporidia, which is functionally consistent with the absence of the alternative oxidase protein in both species.

## Methods

### Characterisation of AOX genes in microsporidia

The *A. locustae* alternative oxidase sequence was retrieved from the GMOD MBL *A. locustae* database and used to design degenerate primers to amplify a fragment of the alternative oxidase gene from *T. hominis, G. plecoglossi and S. lophii* (Forward 5′-GAAACWGTWGCWGCWGTNCCNGG-3′, Reverse 5′-ATWGCTTCTTCTTCNAKRTANCCNAC-3′). Degenerate PCR was carried out on DNA from *E. cuniculi* to exclude the possibility that the AOX gene is present in the genome within the subtelomeric regions that have not been fully assembled [6]. This gave negative results. Negative degenerate PCR results were found for *Brachiola algerae* and *Edhazardia aedis*. The full-length gene was amplified from *T. hominis* DNA and RNA obtained from purified spores from cultures maintained in rabbit kidney cells at Rutgers, State University of New Jersey. The 5′ prime end of the gene was amplified using RLM-RACE using primers designed from within the fragment amplified by degenerate PCR. The first round of PCR yielded a product truncated at the 5′ end. Primers were then designed from within that fragment to obtain the presumed full-length gene. A splinkerette strategy was used to obtain 3′ end of the gene [38]. Amplified PCR products were cloned using the TOPO TA cloning system (Invitrogen) and sequenced using Big Dye 3.2 (ABI). Mitochondrial transit peptides were predicted using Mitoprot I [46], Predotar [47], and TargetP 1.1 [48]. (New sequences are deposited in the GenBank Database under the accession numbers GU221909-GU221911).

### Heterologous expression in yeast

DNA fragments corresponding to *A. locustae* and *T. hominis* AOX open reading frames were amplified by PCR by using primers that generated in-frame restriction sites. PCR products were cloned upstream of green fluorescent protein (GFP)-S65T under the control of the *MET25* promoter [49] for analysis by confocal or fluorescence microscopy. Constructs were then transformed into the diploid yeast strain JK9-3da/a (*leu2-3,122/leu2-3,122 ura3-52/ura3-52 rme1/rme1 trp1/trp1 his4/his4* GAL+/GAL+ HMLa/HMLa), and plated on uracil and methionine deficient SD plates (2% (w/v) agar, 2% (w/v) glucose and 0.67% (w/v) yeast nitrogen base supplemented with the relevant amino acids). Positive colonies were grown overnight in SD medium lacking uracil and methionine and stained with MitoTracker (MitoTracker Red CM-H_2_XRos) according to the manufacturer's protocol (Molecular Probes). Yeast cells were visualized using the Zeiss meta confocal microscope.

### Western blot analysis

Separation of yeast mitochondrial proteins on non-reducing SDS-polyacrylamide gels, transfer to nitrocellulose membranes, and detection of AOX protein using monoclonal antibodies raised against the *S. guttatum* AOX [50] was performed as described previously [51].

### Functional assay

The *A. locustae* and *T. hominis* gene sequences were amplified using Phusion High-Fidelity Taq (New England Biolabs) and cloned into the pet14b expression vector. Both constructs were used to transform *E. coli* strain C41, which is especially suited to the expression of transmembrane proteins. Bacterial membranes were prepared using 2.5 L Luria broth cultures, adapted from Berthold [52] and as described in detail by Crichton *et al* 2009 [53]. Flasks containing Luria Broth, 0.02% glucose, 0.002% FeSO_4_ and 50 µgml^−1^ ampicillin were inoculated with 10 mlL^−1^ starter culture, and incubated at 37°C for 4 hours. The temperature was reduced to 18°C, and the cultures were incubated for one hour prior to induction with 100 µM IPTG. After induction, the cultures were incubated for 18 hours at 18°C. Cells were then harvested using centrifugation at 11,000×g for 10 minutes. After initial centrifugation, cells were resuspended in 60 mM Tris-HCl (pH 7.5), 5 mM DTT, 300 mM NaCl and 0.1M PMSF and then sonicated for 8 minutes at 14 microns. After sonication, cell debris was removed by centrifugation at 12,000×g for 15 minutes, and clear supernatant was further refined by a 2-hour ultracentrifugation step at 200,000×g. Pellets from final spin were resuspended in 60 mM Tris-HCl (pH 7.5), 5 mM DTT, 300 mM NaCl and used for subsequent gel and assay analysis. Ubiquinol oxidase activity (AOX activity) was measured by recording the absorbance change of ubiquinol-1 at 278 nm (Cary UV/vis -400 Scan spectrophotometer). Reactions were started by the addition of ubiquinol-1 (final concentration 150 µM, ε278 = 15,000 M^−1^cm^−1^) after 2 min preincubation at 25°C in the presence of rAlAOX and rThAOX in 50 mM Tris-HCl (pH 7.4). Endogenous ubiquinol activities were inhibited by inclusion of 1 µM antimycin A, 2 µM myxothiazol and 1 mM potassium cyanide in the assay medium.

### Phylogenetic analysis

The *A. locustae* and *T. hominis* AOX amino acid sequences were aligned to 47 diverse proteins sequences with representatives from animal, kinetoplastid, fungal, heterokont, plant and proteobacterial lineages. Sequences were aligned using ClustalW [54] and manually edited and masked. The alignment was analysed using Modelgenerator to select an appropriate model for amino acid change [55]. Phylogenetic trees were inferred using MrBayes 3 [56] with a Blosum62 matrix and with 2 runs each of 1000000 generations carried out on the freely available Bioportal (www.bioportal.uio.no). A burn-in of 400 trees was removed from each run and a consensus created from remaining trees. Five hundred bootstrapped data matrices were also analysed by maximum likelihood using PhyML 3.0 [57] with a JTT model of amino acid change and an estimated gamma parameters with four rate categories of amino-acid change. A second alignment restricted to the conserved area amplified by degenerate PCR from *S. lophii*, *G. plecoglossi* was also analysed. Trees were inferred and 100 bootstrap datasets analysed from this short alignment using PhyML, using the parameters described above. The SSU rRNA backbone phylogeny was based on available SSU sequences from NCBI, which were aligned using ClustalW, manually edited and masked and analysed using PhyML 3.0 with a JC69 nucleotide substitution model with estimated gamma parameter and 4 categories of rate change.

## References

[1] Didier ES, Weiss LM (2006). Microsporidiosis: current status.. Curr Opin Infect Dis.

[2] Keeling PJ, Doolittle WF (1996). Alpha-tubulin from early-diverging eukaryotic lineages and the evolution of the tubulin family.. Mol Biol Evol.

[3] Hirt RP, Logsdon JM, Healy B, Dorey MW, Doolittle WF (1999). Microsporidia are related to Fungi: evidence from the largest subunit of RNA polymerase II and other proteins.. Proc Natl Acad Sci U S A.

[4] Edlind TD, Li J, Visvesvara GS, Vodkin MH, McLaughlin GL (1996). Phylogenetic analysis of beta-tubulin sequences from amitochondrial protozoa.. Mol Phylogenet Evol.

[5] Thomarat F, Vivares CP, Gouy M (2004). Phylogenetic analysis of the complete genome sequence of *Encephalitozoon cuniculi* supports the fungal origin of microsporidia and reveals a high frequency of fast-evolving genes.. J Mol Evol.

[6] Katinka MD, Duprat S, Cornillot E, Metenier G, Thomarat F (2001). Genome sequence and gene compaction of the eukaryote parasite *Encephalitozoon cuniculi*.. Nature.

[7] Beznoussenko GV, Dolgikh VV, Seliverstova EV, Semenov PB, Tokarev YS (2007). Analogs of the Golgi complex in microsporidia: structure and avesicular mechanisms of function.. J Cell Sci.

[8] Williams BA, Hirt RP, Lucocq JM, Embley TM (2002). A mitochondrial remnant in the microsporidian *Trachipleistophora hominis*.. Nature.

[9] Weidner E, Findley AM, Dolgikh V, Sokolova J, Wittner M, Weiss LM (1999). Microsporidian biochemistry and physiology.. The Microsporidia and Microsporidiosis.

[10] Tsaousis AD, Kunji ER, Goldberg AV, Lucocq JM, Hirt RP (2008). A novel route for ATP acquisition by the remnant mitochondria of *Encephalitozoon cuniculi*.. Nature.

[11] Richards TA, Hirt RP, Williams BA, Embley TM (2003). Horizontal gene transfer and the evolution of parasitic protozoa.. Protist.

[12] Williams BA, Cali A, Takvorian PM, Keeling PJ (2008). Distinct localization patterns of two putative mitochondrial proteins in the microsporidian *Encephalitozoon cuniculi*.. J Eukaryot Microbiol.

[13] Goldberg AV, Molik S, Tsaousis AD, Neumann K, Kuhnke G (2008). Localization and functionality of microsporidian iron-sulphur cluster assembly proteins.. Nature.

[14] Larsson C, Pahlman IL, Ansell R, Rigoulet M, Adler L (1998). The importance of the glycerol 3-phosphate shuttle during aerobic growth of *Saccharomyces cerevisiae*.. Yeast.

[15] Slamovits CH, Fast NM, Law JS, Keeling PJ (2004). Genome compaction and stability in microsporidian intracellular parasites.. Curr Biol.

[16] Chaudhuri M, Ott RD, Hill GC (2006). Trypanosome alternative oxidase: from molecule to function.. Trends Parasitol.

[17] Suzuki T, Hashimoto T, Yabu Y, Kido Y, Sakamoto K (2004). Direct evidence for cyanide-insensitive quinol oxidase (alternative oxidase) in apicomplexan parasite *Cryptosporidium parvum*: phylogenetic and therapeutic implications.. Biochem Biophys Res Commun.

[18] Affourtit C, Albury MS, Crichton PG, Moore AL (2002). Exploring the molecular nature of alternative oxidase regulation and catalysis.. FEBS Lett.

[19] Moore AL, Albury MS (2008). Further insights into the structure of the alternative oxidase: from plants to parasites.. Biochem Soc Trans.

[20] Albury MS, Elliott C, Moore AL (2009). Towards a structural elucidation of the alternative oxidase in plants.. Physiol Plant.

[21] Stenmark P, Nordlund P (2003). A prokaryotic alternative oxidase present in the bacterium *Novosphingobium aromaticivorans*.. FEBS Lett.

[22] Vanlerberghe GC, McIntosh L (1997). Alternative oxidase: From gene to function.. Annu Rev Plant Phys.

[23] Jarmuszkiewicz W, Wagner AM, Wagner MJ, Hryniewiecka L (1997). Immunological identification of the alternative oxidase of *Acanthamoeba castellanii* mitochondria.. FEBS Lett.

[24] Stechmann A, Hamblin K, Perez-Brocal V, Gaston D, Richmond GS (2008). Organelles in *Blastocystis* that blur the distinction between mitochondria and hydrogenosomes.. Curr Biol.

[25] McDonald AE, Vanlerberghe GC, Staples JF (2009). Alternative oxidase in animals: unique characteristics and taxonomic distribution.. J Exp Biol.

[26] Gardner MJ, Hall N, Fung E, White O, Berriman M (2002). Genome sequence of the human malaria parasite *Plasmodium falciparum*.. Nature.

[27] Roberts CW, Roberts F, Henriquez FL, Akiyoshi D, Samuel BU (2004). Evidence for mitochondrial-derived alternative oxidase in the apicomplexan parasite *Cryptosporidium parvum*: a potential anti-microbial agent target.. Int J Parasitol.

[28] Eisen JA, Coyne RS, Wu M, Wu D, Thiagarajan M (2006). Macronuclear genome sequence of the ciliate *Tetrahymena thermophila*, a model eukaryote.. PLoS Biol.

[29] Atteia A, van Lis R, van Hellemond JJ, Tielens AGM, Martin W (2004). Identification of prokaryotic homologues indicates an endosymbiotic origin for the alternative oxidases of mitochondria (AOX) and chloroplasts (PTOX).. Gene.

[30] Joseph-Horne T, Hollomon DW, Wood PM (2001). Fungal respiration: a fusion of standard and alternative components.. Biochim Biophys Acta.

[31] Akiyoshi DE, Morrison HG, Lei S, Feng X, Zhang Q (2009). Genomic survey of the non-cultivatable opportunistic human pathogen, *Enterocytozoon bieneusi*.. PLoS Pathog.

[32] Cornman RS, Chen YP, Schatz MC, Street C, Zhao Y (2009). Genomic analyses of the microsporidian *Nosema ceranae*, an emergent pathogen of honey bees.. PLoS Pathog.

[33] Nihei C, Fukai Y, Kita K (2002). Trypanosome alternative oxidase as a target of chemotherapy.. Biochim Biophys Acta.

[34] Kita K, Nihei C, Tomitsuka E (2003). Parasite mitochondria as drug target: Diversity and dynamic changes during the life cycle.. Curr Med Chem.

[35] Didier ES (1997). Effects of albendazole, fumagillin, and TNP-470 on microsporidial replication in vitro.. Antimicrob Agents Chemother.

[36] Didier PJ, Phillips JN, Kuebler DJ, Nasr M, Brindley PJ (2006). Antimicrosporidial activities of fumagillin, TNP-470, ovalicin, and ovalicin derivatives in vitro and in vivo.. Antimicrob Agents Chemother.

[37] Minagawa N, Yabu Y, Kita K, Nagai K, Ohta N (1997). An antibiotic, ascofuranone, specifically inhibits respiration and in vitro growth of long slender bloodstream forms of *Trypanosoma brucei brucei*.. Mol Biochem Parasitol.

[38] Qureshi SJ, Porteous DJ, Brookes AJ (1994). Alu-based vectorettes and splinkerettes. More efficient and comprehensive polymerase chain reaction amplification of human DNA from complex sources.. Genet Anal Tech Appl.

[39] Moore AL, Carre JE, Affourtit C, Albury MS, Crichton PG (2008). Compelling EPR evidence that the alternative oxidase is a diiron carboxylate protein.. Biochim Biophys Acta.

[40] Umbach AL, Ng VS, Siedow JN (2006). Regulation of plant alternative oxidase activity: a tale of two cysteines.. Biochim Biophys Acta.

[41] Vossbrinck CR, Debrunner-Vossbrinck BA (2005). Molecular phylogeny of the Microsporidia: ecological, ultrastructural and taxonomic considerations.. Folia Parasitol (Praha).

[42] Fukai Y, Nihei C, Kawai K, Yabu Y, Suzuki T (2003). Overproduction of highly active trypanosome alternative oxidase in *Escherichia coli* heme-deficient mutant.. Parasitology International.

[43] Williams BA, Lee RC, Becnel JJ, Weiss LM, Fast NM (2008). Genome sequence surveys of *Brachiola algerae* and *Edhazardia aedis* reveal microsporidia with low gene densities.. BMC Genomics.

[44] Albury MS, Affourtit C, Crichton PG, Moore AL (2002). Structure of the plant alternative oxidase. Site-directed mutagenesis provides new information on the active site and membrane topology.. J Biol Chem.

[45] Nina M, Berneche S, Roux B (2000). Anchoring of a monotopic membrane protein: the binding of prostaglandin H2 synthase-1 to the surface of a phospholipid bilayer.. Eur Biophys J.

[46] Claros MG (1995). MitoProt, a Macintosh application for studying mitochondrial proteins.. Comput Appl Biosci.

[47] Small I, Peeters N, Legeai F, Lurin C (2004). Predotar: A tool for rapidly screening proteomes for N-terminal targeting sequences.. Proteomics.

[48] Emanuelsson O, Nielsen H, Brunak S, von Heijne G (2000). Predicting subcellular localization of proteins based on their N-terminal amino acid sequence.. J Mol Biol.

[49] George R, Beddoe T, Landl K, Lithgow T (1998). The yeast nascent polypeptide-associated complex initiates protein targeting to mitochondria in vivo.. Proc Natl Acad Sci U S A.

[50] Elthon TE, Nickels RL, McIntosh L (1989). Monoclonal antibodies to the alternative oxidase of higher plant mitochondria.. Plant Physiol.

[51] Albury MS, Dudley P, Watts FZ, Moore AL (1996). Targeting the plant alternative oxidase protein to *Schizosaccharomyces pombe* mitochondria confers cyanide-insensitive respiration.. J Biol Chem.

[52] Berthold DA, Andersson ME, Nordlund P (2000). New insight into the structure and function of the alternative oxidase.. Biochim Biophys Acta.

[53] Crichton PG, Affourtit C, Albury MS, Elliot C, Wei DM (2009). Mutagenesis of the plant alternative oxidase reveals features important for oxygen binding and catalysis, and traps a stable ubisemiquinone in the ubiquinone binding site.. Biochim Biophys Acta: In press.

[54] Thompson JD, Higgins DG, Gibson TJ (1994). CLUSTAL W: improving the sensitivity of progressive multiple sequence alignment through sequence weighting, position-specific gap penalties and weight matrix choice.. Nucleic Acids Res.

[55] Keane TM, Creevey CJ, Pentony MM, Naughton TJ, McInerney JO (2006). Assessment of methods for amino acid matrix selection and their use on empirical data shows that ad hoc assumptions for choice of matrix are not justified.. BMC Evol Biol.

[56] Huelsenbeck JP, Ronquist F (2001). MRBAYES: Bayesian inference of phylogenetic trees.. Bioinformatics.

[57] Guindon S, Gascuel O (2003). A simple, fast, and accurate algorithm to estimate large phylogenies by maximum likelihood.. Syst Biol.

